# Digital Health Information as Friend or Foe: The Question of (Mis)Trust Among Older Internet Users

**DOI:** 10.1093/geroni/igaa057.1322

**Published:** 2020-12-16

**Authors:** Gul Seckin, Patricia Campbell, Megan Lawson

**Affiliations:** 1 University of North Texas, Hickory Creek, Texas, United States; 2 University of North Texas, Denton, United States

## Abstract

Gathering health information is among the major motivations for getting online among older adults who want to be better prepared with knowledge to manage their health and personal care. Prior research also showed significant gender differences in health-related use of the Internet. This research examined the effect of Internet use for health information on (a) mistrust of physician, (b) empowerment, (c) self-care, and (d) worry and/or anxiety. The sample (N=710; Mean= 48.82, SD=16.43) was randomly drawn from a national probability-based online panel. We performed gender-stratified sub-sample analyses of older respondents (age ≥60, N= 194). Hierarchical linear regression analyses showed that there is a negative association between older age and feeling empowered because of using the internet for health information (β = -.23, p < .05) and a positive association between older age and mistrust of diagnosis and/or treatment of physician (β = .19, p < .05). Study respondents did not report better self-care as a result of obtaining information from the Internet (β = -.15, p > .05). Lastly, older adults reported less worry and/or anxiety because of information stumbled upon the Internet that may not be accurate (β = -.25, p < .05). Sub-sample analyses showed that there are gender differences. Particularly, older men reported greater mistrust (β = .32, p < .05), and less worry (β = -.44, p ≤ .01) while these associations were not significant among older women. Results call for examination of the synergy of age and gender in perceived benefits of health-related Internet use.

